# Azaphenanthrene Alkaloids with Antitumoral Activity from *Anaxagorea dolichocarpa* Sprague & Sandwith (Annonaceae)

**DOI:** 10.3390/molecules16087125

**Published:** 2011-08-22

**Authors:** Ana Silvia Suassuna Carneiro Lúcio, Jackson Roberto Guedes da Silva Almeida, José Maria Barbosa-Filho, João Carlos Lima Rodrigues Pita, Marianna Vieira Sobral Castello Branco, Margareth de Fátima Formiga Melo Diniz, Maria de Fátima Agra, Emidio V.L. da-Cunha, Marcelo Sobral da Silva, Josean Fechine Tavares

**Affiliations:** 1 Laboratory of Pharmaceutical Technology, Federal University of Paraiba, 58051-900, João Pessoa, PB, Brazil; Email: asilviasuassuna@gmail.com (A.S.S.C.L.); jbarbosa@ltf.ufpb.br (J.M.B.-F.); joaocpita@yahoo.com.br (J.C.L.R.P.); mariannavbs@gmail.com (M.V.S.C.B.); margareth@ltf.ufpb.br (M.F.F.M.D.); agramf@ltf.ufpb.br (M.F.A.); marcelosobral@ltf.ufpb.br (M.S.S.); 2 Academic Collegiate of Pharmaceutical Sciences, Federal University of Sao Francisco Valley, P.O. Box 252, 56306-410, Petrolina, PE, Brazil; Email: jackson.guedes@univasf.edu.br; 3 State University of Paraíba, Department of Pharmacy, 58100-000, Campina Grande, PB, Brazil; Email: emidio@ltf.ufpb.br

**Keywords:** Annonaceae, *Anaxagorea dolichocarpa*, azaphenanthrene alkaloid, antitumor activity

## Abstract

Phytochemical investigation of *Anaxagorea dolichocarpa* Sprague & Sandwith led to isolation of three azaphenanthrene alkaloids: eupolauramine, sampangine and imbiline 1. Their chemical structures were established on the basis of spectroscopic data from IR, HR-ESI-MS, NMR (including 2D experiments) and comparison with the literature. Sampangine and imbiline 1 are being described in the *Anaxagorea* genus for the first time. Eupolauramine and sampangine show concentration-dependent antitumoral activity in leukemic cells K562 with IC_50_ of 18.97 and 10.95 µg/mL, respectively.

## 1. Introduction

The family Annonaceae comprises 135 genera, distributed mainly in the tropical and subtropical regions of the World [1]. Phytochemical studies of the family’s species have noted the presence of acetogenins [2,3], alkaloids [4,5] and terpenoids [6], with the predominant class being alkaloid secondary metabolites, especially aporphine alkaloids [7].

The genus *Anaxagorea* includes about 26 species distributed throughout North, Central and South America [8]. Among the known species, only five (*A. dolichocarpa*, *A. brevipes*, *A. luzonensis*, *A. clavata* and *A. prinoides*) have been studied phytochemically, from which lignoids [9], xantones [10], flavonoids [11] and alkaloids [12] were isolated.

*Anaxagorea dolichocarpa* shows a wide geographical distribution, occurring from Costa Rica to Bolivia and in Brazil, in the states of Amapá, Amazonas, Acre, Rondônia, Goiás, Maranhão, Paraíba, Pernambuco, Bahia and Rio de Janeiro. It always grows in humid forests [13]. Previous studies on this species reported the isolation of the aporphine alkaloids anaxagoreine and asimilobine [12], the composition of the fruits’ essential oil [14] and an antimicrobial activity test against *Staphylococcus aureus* [15].

Since there is very little phytochemical literature on this species and as it is potentially a good source of bioactive compounds, we decided to expand its phytochemical investigation. We report here the isolation and chemical characterization of three azaphenanthrene alkaloids: eupolauramine (**1**), sampangine (**2**) and imbiline 1 (**3**) isolated from *A. dolichocarpa* for the first time. The *in vitro* antitumor activity of eupolauramine and sampangine was also evaluated.

## 2. Results and Discussion

**Figure 1:**
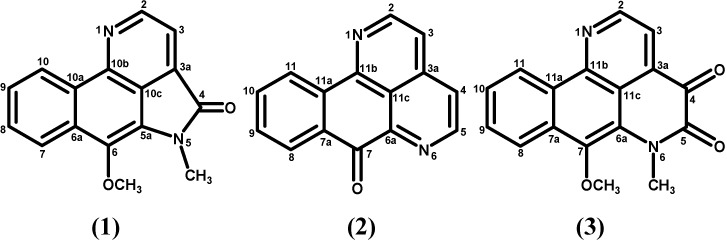
Alkaloids isolated from *Anaxagorea dolichocarpa*.

Phytochemical investigation of *Anaxagorea dolichocarpa* led to the isolation of three azaphenantrene alkaloids: eupolauramine (**1**), sampangine (**2**) and imbiline 1 (**3**), a unique structural series obtained from the plant (Figure 1). The interesting structure of these compounds, together with their rare occurrence makes the study of their pharmacology and the development of potential synthetic routes to these compounds interesting [16,17]. The structural elucidation of these alkaloids was carried out based on their spectroscopic data, mainly 1D and 2D NMR, and also comparison with the literature data [18,19,20]. This work reports the first 2D NMR data for eupolauramine (**1**) and sampangine (**2**). Compound **1** was previously isolated from the species *Eupomatia laurina* of the family Eupomatiaceae [20] and is being reported for the first time in the family Annonaceae. Compound **2** has been previously reported from *Cananga odorata* and *Cleistopholis patens*, both from the family Annonaceae [16]. Compound **3** was already isolated from *Duguetia hadrantha* (Annonaceae), *Eupomatia laurina* and *E. bennettii* (Eupomatiaceae) [16]. Compounds **2** and **3** are described for the first time as isolated from the genus *Anaxagorea*. Compound **2** holds special interest for its relevant pharmacological activities: antifungal [21], anti-malarial [18], antibacterial [22] antitumoral against HL-60 cells (myeloid acute leukemia) [23] and also against human malignant melanoma cells [18].

The use of natural products as anticancer agents has a long history that began with folk medicine and through the years has been incorporated into allopathic medicine. Many drugs currently used in chemotherapy were isolated from plant species or derived from a natural prototype [24]. The study of natural products represents the most successful strategy for discovering new drugs for anticancer therapy [25]. 

**Figure 2:**
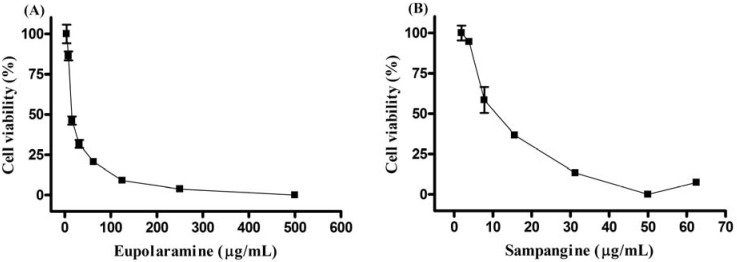
Celular viability after the treatment with the alkaloids eupolauramine (**A**) and sampangine (**B**).

The *in vitro* effects of eupolauramine (**1**) and sampangine (**2**) against the K562 cell line were determined in three experiments and in quadruplicate (compound **3** could not be tested due to the small amount available). Both compounds exhibited concentration-dependent inhibitory effect on the proliferation of K562 cells. The IC_50_ values were 18.97 (17.06–21.10) μg/mL and 10.95 (10.15–11.80) μg/mL respectively, as evaluated by the MTT reduction assay (Figure 2).

## 3. Experimental

### 3.1. General

The mass spectra were obtained in positive ion mode through the (+) electrospray technique using a Bruker Microtof II high resolution mass spectrometer. The ^1^H and ^13^C 1D and 2D NMR experiments were recorded on a Varian-NMR-System 500 [500 MHz (^1^H) and 125 MHz (^13^C)]. CDCl_3_ was the solvent used for all of the NMR experiments and the solvent peaks were used to calibrate the equipment. For column chromatography, Merck 60 silica gel (0,063–0,200 mm) was used. For the preparative and analytical TLC 60 PF_254_ ART 7749 silica gel (Merck) was used. The compounds in analysis were observed through the use of UV light (254 e 366) and after spraying with Dragendorff’s reagent. 

### 3.2. Plant material

The stem bark of *Anaxagorea dolichocarpa* was collected in the city of Santa Rita, State of Paraíba, Brazil, in February 2006. It was identified by Prof. Maria de Fátima Agra from the Botany Laboratory of the Federal University of Paraiba. A voucher specimen was deposited at the Herbarium Prof. Lauro Pires Xavier (JPB), of the Federal University of Paraiba, with the number Agra & Góes 5543.

### 3.3. Extraction and isolation procedures

The dried and pulverized stem bark (2,000 g) was subjected to maceration with 95% EtOH (3 × 5 L) for 72 hours at room temperature. The EtOH solution was concentrated under vacuum, yielding 64 g of crude EtOH extract (CEE), which was suspended in MeOH-H_2_O (3:7) and partitioned with hexane (3,000 mL), CHCl_3_ (1,500 mL) and ethyl acetate (1,500 mL). After removal of the solvents the CHCl_3_ (14.0 g) and hexane (11.0 g) extracts were subjected to column chromatography over silica gel using hexane, CHCl_3_ and MeOH, either pure or in binary mixtures. Thus, from the CHCl_3_ extract column 183 fractions were obtained, which were analyzed by analytical TLC and combined according to their chromatographic profiles into 23 groups. The group represented by fractions 12–24 was further purified by silica gel preparative TLC, using dichloromethane-acetonitrile (97:3) as eluent [12], yielding compound **1** (0.023 g). Similarly, from the hexane extract column 277 fractions were obtained and combined into 22 groups. The groups represented by the fractions 135–148 and 198–205 were purified by silica gel preparative TLC using as eluents pure CHCl_3_ and CHCl_3_-MeOH, (99:1), respectively, to yield compounds **2** (0.020 g) and **3** (0.010 g).

*Eupolauramine* (**1**): Orange crystals. Mp 190–193 °C (lit. [19] Mp 191–192.5 °C). HRMS *m/z* 288.2845 [M + Na]^+^ (C_16_H_12_N_2_O_2_Na). ^1^H-NMR: δ_H_ 3.73 (s, MeN-5), 4.06 (s, MeO-6), 7.67 (ddd, *J* = 8.5, 7.5 and 1.5 Hz, H-9), 7.74 (ddd, *J* = 8.8, 7.0 and 1.5 Hz, H-8), 7.90 (d, *J* = 4.5 Hz, H-3), 8.13 (dd, *J* = 8.0 and 0.5 Hz, H-7), 9.00 (dd, *J* = 8.5 and 1.0 Hz, H-10), 9.15 (d, *J* = 4.5 Hz, H-2). ^13^C-NMR: δ_C_ 28.4 (MeN-5), 63.4 (MeO-6), 117.1 (C-3), 122.6 (C-10c), 122.7 (C-7), 124.2 (C-10), 124.9 (C-5a), 126.6 (C-9), 129.3 (C-8), 130.0 (C-10a), 132.5 (C-6a), 133.1 (C-3a), 137.7 (C-6), 142.9 (C-10b), 150.4 (C-2), 167.0 (C-4). HMBC: H-2/C-3, C-3a, C-4, C-10b; H-3/C-2, C-4, C-10c; H-7/C-6, C-9, C-10a; H-8/C-6a, C-7, C-10; H-9/C-7, C-10, C-10a; H-10/C-6a, C-8, C-10b; MeO-6/C-6; MeN-5/C-4, C-5a. COSY: H-2/H-3, H-7/H-8, H-9/H-10.

*Sampangine* (**2**): Yellow crystals. Mp 212–215 °C (lit. [16] Mp 215–217 °C). HRMS *m/z* 255.0495 [M + Na]^+^ (C_15_H_8_N_2_ONa). ^1^H-NMR: δ_H_ 7.68 (ddd, *J* = 8.0, 8.5 and 1.0 Hz, H-9), 7.71 (d, *J* = 6.0 Hz, H-3), 7.82 (ddd, *J* = 8.0, 8.5 and 1.5 Hz, H-10), 7.91 (d, *J* = 5.5 Hz, H-4), 8.45 (dd, *J* = 7.5 and 1.0 Hz, H-8), 8.82 (dd, *J* = 8.0 and 0.5 Hz, H-11), 8.88 (d, *J* = 6.0 Hz, H-2), 9.12 (d, *J* = 5.5 Hz, H-5). ^13^C-NMR: δ_C_ 119.1 (C-3), 119.8 (C-11c), 123.5 (C-4), 125.4 (C-11), 128.5 (C-8), 131.4 (C-9), 132.4 (C-7a), 134.7 (C-10), 135.5 (C-11a), 138.7 (C-3a), 147.4 (C-2), 148.0 (C-6a), 148.5 (C-5), 151.3 (C-11b), 182.0 (C-7). HMBC: H-2/C-3c, C-3a, C-11b; H-3/C-2, C-4, C-11c; H-4/C-3, C-5, C-11c; H-5/C-3a, C-4, C-6a; H-8/C-10, C-11a; H-9/C-7a, C-11; H-10/C-8, C-11a; H-11/C-7a, C-9. COSY: H-2/H-3, H-4/H-5, H-8/H-9, H-9/H-8,H-10; H-10/H-9,H-11; H-11/H-10.

*Imbiline 1* (**3**): Red crystals. Mp 215–217 °C (lit. [20] Mp 212–214 °C). ^1^H-NMR: δ_H_ 3.95 (s, MeN-6), 4.00 (s, MeO-7), 7.77 (ddd, *J* = 8.0, 7.0 and 1.0 Hz, H-10), 7.81 (ddd, *J* = 8.5, 7.5 and 1.5 Hz, H-9), 8.23 (dd, *J* = 9.5 and 1,5 Hz, H-8), 8.27 (d, *J* = 4.5 Hz, H-3), 9.21 (d, *J* = 1.5 Hz, H-11), 9.23 (d, *J* = 5.5 Hz, H-2). ^13^C-NMR: δ_C_ 35.9 (MeN-6), 63.0 (MeO-7), 118.3 (C-11c), 119.3 (C-3), 122.6 (C-6a), 122.8 (C-8), 125.2 (C-11), 128.5 (C-10), 129.7 (C-11a), 130.4 (C-9), 130.7 (C-7a), 132.3 (C-3a), 144.9 (C-7), 145.1 (C-11b), 148.2 (C-2), 157.6 (C-5), 176.5 (C-4). HMBC: H-2/C-3, C-3a, C-11b; H-3/C-2, C-11c; H-8/C-7, C-9, C-10, C-11a; H-9/C-7a, C-11; H-10/C-8, C-9, C-11a; H-11/C-7a, C-9, C-11b. COSY: H-2/H-3, H-8/H-9, H-10/H-11.

### 3.4. Evaluation of “in vitro” antitumor activity

The human leukemic strain K562 (Chronic Myeloid Leukemia) was cultivated in a RPMI-1640 (Nutricell®) media supplemented with 2 g/L NaHCO_3_ (Sigma-Aldrich), 2 mM L-glutamine (Nutricell®), 100 UI/mL of penicillin, 100 μg/mL de streptomycin (Sigma-Aldrich) and 10% bovine fetal serum (Nutricell). The K562 cells (3 × 10^5^ cell/mL) were arranged in 96 wells with different concentrations (0–500 μg/mL) of eupolauramine and sampangine and incubated at 37 °C and 5% CO_2_ for 72 h. The compounds were initially dissolved in dimethylsulfoxide (DMSO) and afterwards complemented with the media. The final DMSO concentration was 0.5%. The cytotoxity was evaluated through the MTT reduction assay, which determines the number of living cells able to reduce the yellow dye 3-(4,5-dimethyl-2-thiazolyl)-2,5-diphenyl-2H-tetrazolium bromide (MTT) to formazan, which has a purple color [26]. In brief, 0.01 mL of the media was added to each well. After incubating for 4 hours, formazan was dissolved in 0.1 mL of EtOH under agitation over a microplate shaker for 10 minutes. Afterwards absorbance was read at 595 nm with the use of a microplate reader (Bio-Rad^®^ Berkeley, CA, USA) for the determination of the 50% inhibition concentration for cell growth (IC_50_).

## 4. Conclusions

Three azaphenanthrene alkaloids were isolated from the stem bark of *A. dolichocarpa*. Both sampangine and imbiline 1 are being reported for the first time in the genus *Anaxagorea* and eupolauramine for the first time in the family Annonaceae. These data contribute to the chemotaxonomy of Annonaceae, and especially for the genus *Anaxagorea*. The alkaloids eupolauramine and sampangine demonstrated antitumor activity against human cells strain K569 showing IC_50_ values of 18.97 (17.06–21.10) μg/mL and 10.95 (10.15–11.80) μg/mL, respectively. The interesting antitumoral activities of these alkaloids suggests great potential in the ongoing search for molecules with activity against cancer.
